# Spatial Distribution of Attentional Modulation at Columnar Resolution in Macaque Area V4

**DOI:** 10.3389/fncir.2016.00102

**Published:** 2016-12-12

**Authors:** Hisashi Tanigawa, Gang Chen, Anna W. Roe

**Affiliations:** ^1^Interdisciplinary Institute of Neuroscience and Technology, Zhejiang UniversityHangzhou, China; ^2^Center for Transdisciplinary Research, Niigata UniversityNiigata, Japan; ^3^Department of Psychology, Vanderbilt UniversityNashville, TN, USA

**Keywords:** spatial attention, intrinsic signal optical imaging, column, normalization model, global signals, mapping signals, cerebral blood volume

## Abstract

Attention to a location in a visual scene affects neuronal responses in visual cortical areas in a retinotopically specific manner. Optical imaging studies have revealed that cortical responses consist of two components of different sizes: the stimulus-nonspecific global signal and the stimulus-specific mapping signal (domain activity). It remains unclear whether either or both of these components are modulated by spatial attention. In this study, to determine the spatial distribution of attentional modulation at columnar resolution, we performed cerebral blood volume (CBV)-based optical imaging in area V4 of monkeys performing a color change detection task in which spatial attention was manipulated. We found that spatial attention enhanced global signals of the hemodynamic responses, but did not affect stimulus-selective domain activities. These results indicate the involvement of global signals in neural processing of spatial attention. We propose that global signals reflect the neural substrate of the normalization pool in normalization models of attention.

## Introduction

Electrophysiological studies have demonstrated that attention directed to a specific spatial location in the visual field (spatial attention) enhances activities of neurons whose receptive fields correspond to the attended location (e.g., Connor et al., 1996, 1997; Treue and Maunsell, 1996; McAdams and Maunsell, 1999; Reynolds et al., 2000; Williford and Maunsell, 2006). The spatial distribution of such attentional modulation can be visualized by functional imaging techniques. Blood oxygenation level-dependent (BOLD) functional MRI (fMRI) revealed that the distribution of attentional modulation in visual cortical areas is organized in a retinotopically specific manner (Tootell et al., 1998; Brefczynski and DeYoe, 1999; Gandhi et al., 1999; Kastner et al., 1999; McMains and Somers, 2004). In addition, voltage-sensitive dye (VSD) imaging revealed that the membrane potentials of a population of neurons, which are thought to primarily reflect synaptic input to these cells, are also enhanced by spatial attention in an additive and stimulus-nonspecific manner (Chen and Seidemann, 2012). The enhancement of these neuronal activities could account for the improved behavioral performance associated with attention (Ress et al., 2000; Cohen and Maunsell, 2010, 2011b).

Intrinsic signal optical imaging (ISOI) is a high-resolution optical analog of fMRI (Fukuda et al., 2006) that can visualize cortical hemodynamics at columnar resolution (~50 μm; Frostig et al., 1990; Bonhoeffer and Grinvald, 1991). Typically, ISOI reveals two different-sized complementary components of stimulus-evoked hemodynamic response in the cortex: the “global signal” and “mapping signal” (Frostig et al., 1990; Malonek and Grinvald, 1996; Vanzetta et al., 2004; Fukuda et al., 2005). The global signal spreads laterally over several millimeters or more in the cortex and is stimulus-nonspecific (not significantly different across stimuli), whereas the mapping signal is localized within a lateral extent of about 0.5 mm, forming a domain-like structure, and is stimulus-specific. For researchers who are interested in mapping stimulus preferences, such as orientation preference, in the cortex and visualizing functional domains (columns), global signals are unnecessary and usually removed by post-processing (e.g., Frostig et al., 1990; Tsunoda et al., 2001; Tanigawa et al., 2010). Even though the global signal is substantially larger than the mapping signal (Frostig et al., 1990; Fukuda et al., 2005), the functional significance of global signals is less clear than that of the mapping signal.

In this study, we used ISOI to examine the spatial distribution of attentional modulation at columnar resolution in area V4 of monkeys performing a color change detection task in which we manipulated spatial attention by cueing the location in a block design. We performed ISOI at 570 nm wavelength (an isosbestic, or equal absorption point of oxy- and deoxyhemoglobin), which emphasizes changes in cerebral blood volume (CBV; Frostig et al., 1990; Malonek et al., 1997). CBV-based hemodynamic signals are well correlated with neuronal activities in the cortex, including spiking activities and local-field-potential (LFP) measurements (Sheth et al., 2003; Nemoto et al., 2004; Niessing et al., 2005; Lima et al., 2014). We visualized maps of global and mapping signals of stimulus-evoked CBV-based hemodynamic responses under different attentional conditions and evaluated how these two types of signals were affected by attention. We found that global signals, but not mapping signals, of CBV-based hemodynamic responses were enhanced by spatial attention. Such stimulus-nonspecific large-scale attentional enhancement may reflect the spatial distribution of the normalization pool in normalization models of attention (Lee and Maunsell, 2009; Reynolds and Heeger, 2009).

## Materials and Methods

### Animal Preparation

Two adult rhesus monkeys (*Macaca mulatta*; monkey M1, male, 7 kg; monkey M2, female, 5 kg) were used for experiments. All procedures were approved by the Vanderbilt Animal Care and Use Committees and conformed to the guidelines of the US National Institutes of Health. Prior to training and imaging, under sterile surgical conditions, each monkey was anesthetized and implanted with a head post for head fixation and a chronic nylon chamber overlying dorsal (D) V4 for optical imaging. The chamber was located on the right hemisphere of M1 and on the right hemisphere of M2. Native dura in the chamber was replaced with a clear artificial dura (Tecoflex, Thermedics Polymer Products). After the surgery, the chamber was sealed with a nylon cap and opened under sterile conditions for image acquisition. The procedures for surgery, anesthesia and chamber maintenance were previously described in detail (Chen et al., 2002; Lu and Roe, 2008; Tanigawa et al., 2010).

### Passive Viewing Task

To determine optimal stimulus parameters for the imaged cortical region, we first carried out optical imaging (described below) in that region while the monkey passively viewed various grating stimuli (passive viewing task). Stimuli were created using ViSaGe (Cambridge Research Systems) and presented on a CRT monitor (100 Hz refresh rate, 800 × 600 pixels, gamma corrected) positioned 122 cm from the eyes. Eye position was monitored with an infrared eye tracker (iView X, SensoMotoric Instruments). The monkey initiated the trial by fixating on a spot (0.15°) on a gray background (26.8 cd m^−2^) and maintained fixation until the spot disappeared (fixation window radius, <0.75°) in order to obtain a juice reward. After a 0.5 s pre-stimulus period, a circular patch of isoluminant red/green or luminance-contrast (100%) white/black drifting sinusoidal gratings (1 cycle/° spatial frequency, 1°/s drift rate, one of four different orientations) was presented for 3.5 s. The average luminance of gratings was identical to the background luminance. The phase and drift direction of gratings were randomized on each trial. By systematically altering the location, size and grating orientation of patch (see Tanigawa et al., 2010), we optimized these parameters so that the stimulus could activate the center portion of the imaged region and visualize at least multiple color- and orientation-preferring domains (described below). In all cases, stimuli closer to the vertical meridian activated regions closer to the lunate sulcus (lu), consistent with known retinotopy in V4 (Gattass et al., 1988). For both M1 and M2, the patch was 3° and 4° in diameter and centered at 5.5° and 5.5° eccentricity, 22.5° counterclockwise and 33.75° clockwise away from the vertical meridian in the lower visual field, respectively. We refer to a stimulus at these locations as the stimulus inside the population receptive field (pRF) of the imaged region, where pRF refers to the region of visual space that activates the recording site (Victor et al., 1994).

### Color Change Detection Task

**Figure 1:**
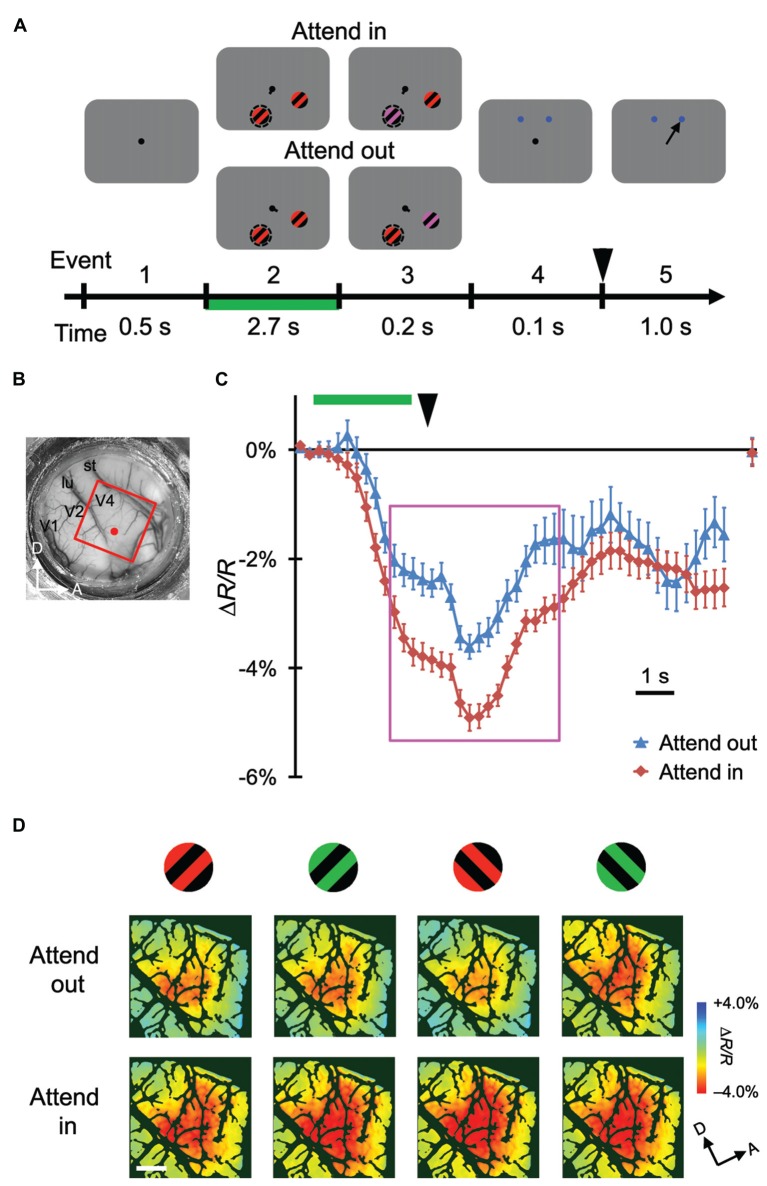
**Stimulus-evoked hemodynamic responses in different attentional conditions. (A)** Sequence and duration of events in a trial of the covert attention task for monkey M1. Broken circles and solid arrows indicate the cued location and correct saccade, respectively, but were not actually present on the monitor. **(B)** A view of the cortical surface of M1, including dorsal V4, through the chamber. The red rectangle indicates the imaged region for this monkey. **(C)** Examples of time courses of average stimulus-evoked reflectance change from the baseline under 570 nm illumination. The sampled site is indicated by a red dot in **(B)**. Error bars represent standard error of the mean (SEM). The rightmost plot indicates the reflectance change from the baseline, measured at the beginning of pre-stimulus period of the subsequent trial. Green horizontal line and black arrowhead indicate the period of stimulus presentation before color change and the timing of disappearance of fixation dot, respectively. These features are also shown in **(A)**. **(D)** The top and bottom panels show maps of reflectance changes evoked by four different stimuli, as indicated on the top, obtained in the imaged region under attend-out (top) and attend-in (bottom) conditions respectively. To make these maps, we averaged the signals from 2 s–6.5 s after the stimulus onset, as indicated by the magenta rectangle in **(C)**, on a pixel-by-pixel basis, and applied blank subtraction to extract stimulus-evoked signals (see “Materials and Methods” Section). Color scale indicates percent change from the baseline. Dark green regions indicate pixels with large cross-trial variability (see “Materials and Methods” Section). A, anterior; D, dorsal; lu, lunate sulcus; st, superior temporal sulcus. Scale bar represents 1 mm.

**Figure 2:**
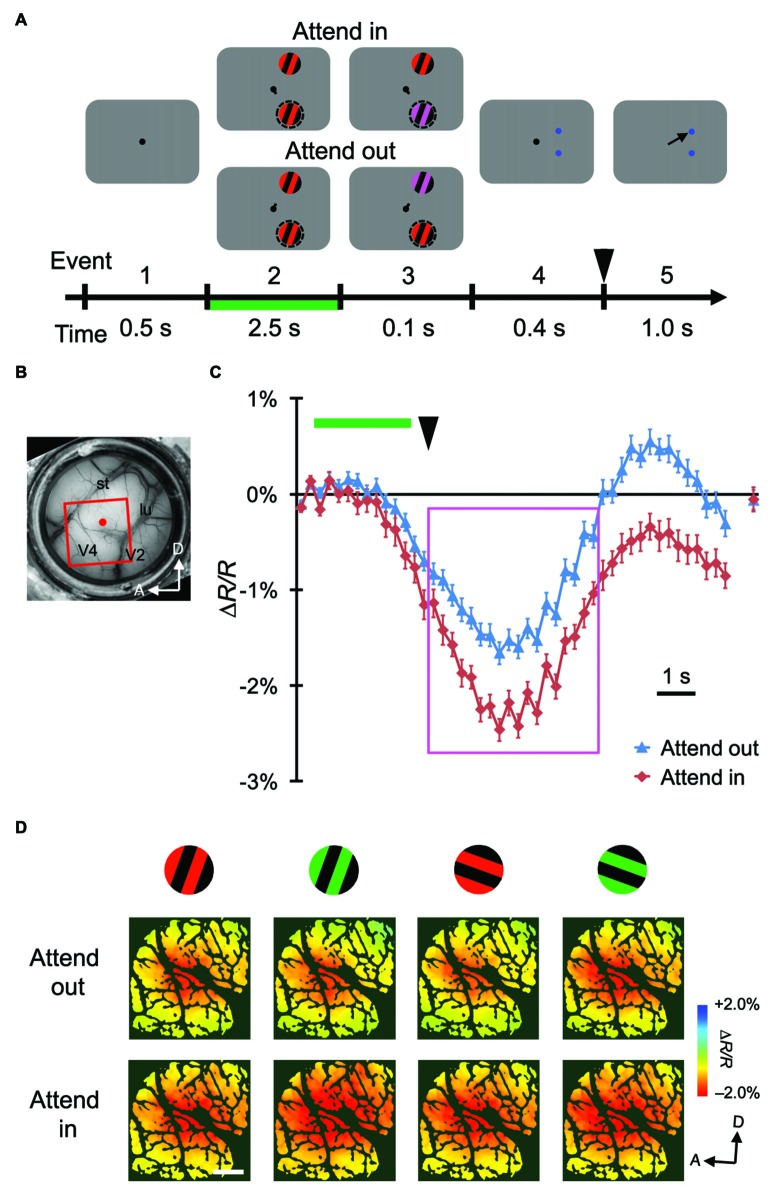
**Stimulus-evoked hemodynamic responses in another monkey. (A,B)** Sequence of the attention task and a view of the cortical surface of monkey M2. The side of imaged hemisphere was opposite to that of M1, and the task sequence and stimuli used were also slightly different. See “Materials and Methods” Section for details. **(C,D)** Time courses and maps of average stimulus-evoked reflectance changes in the imaged region of M2. To make the maps in **(D)**, we averaged the signals from 3 s–7.5 s after stimulus onset, as indicated by the magenta rectangle in **(C)**, on a pixel-by-pixel basis. Conventions are the same as Figure 1.

To study attentional modulation in V4, we employed a color change detection task (Figures 1A, 2A). The stimulus parameters in the task were same as those in the fixation task described above, except for the following details. The task consisted of five events. The monkey initiated the trial by fixating on a fixation spot. After a 0.5 s pre-stimulus period (event 1), two circular patches of red/black or green/black drifting sinusoidal gratings (100% luminance contrast; tilted 45° or 135° counterclockwise from the horizontal for M1, 70° or 160° for M2) were presented (event 2). The two patches were identical except for their locations. One of them was positioned inside the pRF of the imaged region. The other stimulus was positioned 90° (for M1) or 112.5° (for M2) counterclockwise away at the same eccentricity, causing no activation in the imaged region. After a specified time from the onset of stimuli, the color of the gratings in one of the stimuli changed to either yellowish or bluish (event 3). More exactly, in the CIE 1931 *xy* chromaticity diagram, the grating color, either red (0.63, 0.34) or green (0.28, 0.61), shifted by a certain amount to either yellow (0.39, 0.53) or blue (0.15, 0.07), maintaining the same luminance. The degree of color shift was determined in the CIE 1976 *u’v’* chromaticity diagram to be close to the monkey’s detection threshold (0.1–0.2). A short time after the onset of color change, both grating stimuli disappeared, followed by a delay period (event 4). Then, the fixation spot also disappeared, and instead two blue target spots (0.15°) appeared for 1 s (event 5). The targets were located at 4.7° (for M1) or 4.5° (for M2) eccentricity, away from the pRF of the imaged region, and separated by 5° (for M1) or 4° (for M2). The monkey was given a juice reward for making a saccade to the correct target within 0.5 s after the target onset. The location of correct target was associated with the type of grating color change (e.g., left target for yellowish change and right target for bluish change). After target spots disappeared (the end of event 5), there was a 10 s interval before the next presentation of a fixation spot. Fixation was not required during this interval. Even though the total length of the task (from events 1–5) was fixed to 4.5 s, there were small differences in the durations of events 3–5 between the two monkeys (see Figures 1A, 2A). This is because we manipulated task difficulty by changing the duration of these events, as well as by changing the degree of color shift of gratings, so that the monkeys performed the task at 80%–90% success rate. Because these parameters were held constant between different attentional conditions for each monkey, differences in these parameters between animals should not affect our conclusions about attentional modulation.

The location of the patch with the color change alternated between two locations in a block design. Blocks consisted of 27 trials. At the beginning of each block, the monkey performed three instruction trials in which only one of the patches was shown; the monkey performed the task on that patch. On subsequent trials within the block, which were used for the analysis, the stimulus at the same location always showed a color change (cued stimulus). The cued location was also indicated by a short line next to the fixation spot, pointing to the location. In approximately 20% of all trials with two patches, the uncued stimulus (distractor) also displayed a color change simultaneously with the cued stimulus, but the type of color change was different from that of the cued stimulus (yellowish or bluish). We used these trials to probe the attention bias for a cued stimulus. Each block contained eight stimulus conditions (two colors, two orientations, and two cued stimulus position) and one blank condition in which the monkey fixated for 3.5 s and was then given a reword but neither grating stimulus nor target spot appeared. These conditions were repeated in a pseudo-random order. Each imaging session consisted of 6–9 blocks.

### Optical Imaging in Awake Monkeys and Data Analysis

The detailed imaging and data analysis methods have been described previously (Chen et al., 2002; Tanigawa et al., 2010). Under 570 nm illumination, images of light reflectance were captured from a portion of dorsal V4 cortex within the imaging chamber using a CCD video camera (504 × 504 pixels, 8 mm × 8 mm; 1M60P, Dalsa) with a tandem lens system focused on the cortical surface; images were digitized using Imager 3001 (12-bit resolution, 4 frames/s, Optical Imaging). Image acquisition started at the onset of the pre-stimulus period (0.5 s) and continued for at least 10 s for each trial that were analyzed. Image frames were analyzed offline using custom software written in MATLAB (Mathworks). For each trial, the average of frames obtained in the pre-stimulus period was subtracted from all frames on a pixel-by-pixel basis, and then the differences were divided by the same average value, to generate maps of reflectance change (*ΔR/R* map). The Δ*R/R* map obtained in the blank condition (described above) in the same block was then subtracted from the Δ*R/R* maps generated in the previous step on a frame-by-frame basis to extract stimulus-evoked signals (blank subtraction)*.* Finally, the *ΔR/R* maps were averaged over a range of frames and across trials in a particular condition (e.g., color, orientation, cued stimulus location) to form the single condition map for that condition. Difference maps between two conditions were obtained by calculating the average difference of Δ*R/R* maps between the conditions. To extract locally evoked reflectance changes (mapping signals: ~0.5 mm) from large-scale changes (global signals: several millimeters or more), each *ΔR/R* map was convolved with a 1.6 mm × 1.6 mm median filter and subtracted from the original map (high-pass filtering) before blank subtraction.

### Statistical Analysis

We used a two-tailed *t*-test and an ANOVA to evaluate a modulation in signal changes among conditions. The *P* value was calculated by these statistics at each pixel (statistical map). For multiple comparison correction, we adopted a cluster-extent based thresholding procedure (see Woo et al., 2014) in which only regions that consisted of at least 200 contiguous pixels with *P* < 0.05, contained a pixel with *P* < 0.001, and reproduced in another session were regarded as regions with significant modulation; regions that did not meet these criteria were excluded. To remove high-spatial-frequency noise from the statistical maps, we smoothed each Δ*R/R* map before analysis using a 200 μm × 200 μm median filter. Signals from pixels on and near large vessels were less reliable because of large trial-by-trial fluctuation, something that occurred even without visual stimulation. To exclude these regions from the analysis, we calculated pixel-wise standard deviation (SD) of blank-condition images across trials. Pixels with large SD (>the upper limit of 95% one-sided confidence interval based on the *χ*^2^ distribution) were eliminated from further analysis (shaded in dark green in the statistical maps).

## Results

We trained two monkeys (M1 and M2) to perform a color change detection task (Figures 1A, 2A). While the monkeys fixated on the central fixation spot on the monitor, two patches of gratings were presented: one inside and one outside the pRF of imaged V4 region. After a certain time from the onset of stimuli, the color of the gratings in one of the patches changed to either yellowish or bluish. The monkeys reported the type of color change (yellowish or bluish) by making a saccade to the target associated with that type. The location of the patch with color change alternated between the two locations and was cued in a block design. For imaging, each monkey performed two sessions of the color change detection task.

To determine whether attention was directed more to the cued patch than the uncued patch (distractor), in approximately 20% of trials, the distractor also exhibited a color change simultaneously with the cued stimulus, but the type of color change was opposite to that of the cued stimulus. When the color change occurred only in the cued patch, the performance on the attention task was 81% correct for M1 and 88% correct for M2 in trials without a fixation break (*n* = 311 trials for M1 and 304 trials for M2 in two imaging sessions per monkey). When the color change also occurred in the uncued patch, the performance dropped significantly, to 63% and 70% for M1 and M2, respectively (*χ*^2^ test, *P* < 0.001), but remained significantly higher than chance (*χ*^2^ test, *P* < 0.05). These results indicate that the monkeys did not completely ignore the distractor, but that they gave priority to the cued patch when the two patches were in conflict. Therefore, we considered that the monkeys allocated more attention to the cued patch than to the distractor. We defined the “attend-in” condition as the cued patch inside the pRF, and the “attend-out” condition as the cued patch outside the pRF. By comparing hemodynamic responses in the attend-in and attend-out conditions, we could estimate attentional effects on the responses. In the following sections, we report results regarding attentional enhancement based on images captured in trials with a correct behavioral response and stable imaging throughout the trial. Trials with imaging containing a large motion artifact due to the animal movement were regarded as less stable, and were excluded from the imaging analysis.

### Attentional Enhancement of Global Signals of Stimulus-Evoked Hemodynamic Responses in V4

Using ISOI at a wavelength of 570 nm, we measured stimulus-evoked CBV-based hemodynamic responses with blank subtraction (i.e., subtracting the response in the no-stimulus condition; see “Materials and Methods” Section) on a pixel-by-pixel basis in a portion of dorsal V4 (Figures 1B, 2B) of monkeys performing the color change detection task. The time courses of the responses were roughly monophasic, as described in other studies (Sheth et al., 2003; Nemoto et al., 2004; Sirotin et al., 2009), with some fluctuations (Figures 1C, 2C), peaking at 4–5 s after the stimulus onset (note that a negative reflectance change indicates an increase of CBV). There was a difference of about 1 s in the response latency between the two monkeys. This difference might have been due to differences in the timing of when the monkeys started to direct their attention to the stimuli. At the sampled sites of both monkeys, responses were enhanced in the attend-in conditions relative to attend-out conditions. Significant attentional enhancements could already be observed just before the timing of the color change in the patch (one imaging frame (0.25 s) prior to the onset of the color change; two-tailed *t* test;* P* < 0.05, *n* = 76 trials for M1 and 135 trials for M2), indicating that the enhancement was initiated voluntarily prior to the color change. After the 10 s inter-trial interval, the hemodynamic response was reduced to almost the baseline and did not significantly differ between two attentional conditions (Figures 1C, 2C; rightmost point; two-tailed *t* test;* P* > 0.05). Because we observed large enhancements around the peak of the response, we calculated the average responses across a time interval (4.5 s) centered at the peak and across trials on a pixel-by-pixel basis for each stimulus and attentional conditions, and used this information to create maps of hemodynamic responses (Figures 1D, 2D). We performed pixel-by-pixel two-way ANOVAs in which one factor was the stimulus type (four combinations of two colors and two orientations) and the other factor was the attentional conditions (attend-in vs. attend-out), and identified regions with significant increases in attend-in vs. attend-out conditions (*P* < 0.05 and the lowest *P* in each region < 0.001, *n* = 76 trials for M1 and 135 trials for M2, and replicated in another imaging session, *n* = 124 trials for M1 and 161 trials for M2). However, there was no significant difference across stimulus type, and no interaction between the two factors. These results suggest that spatial attention enhances hemodynamic responses in a stimulus-nonspecific manner.

**Figure 3:**
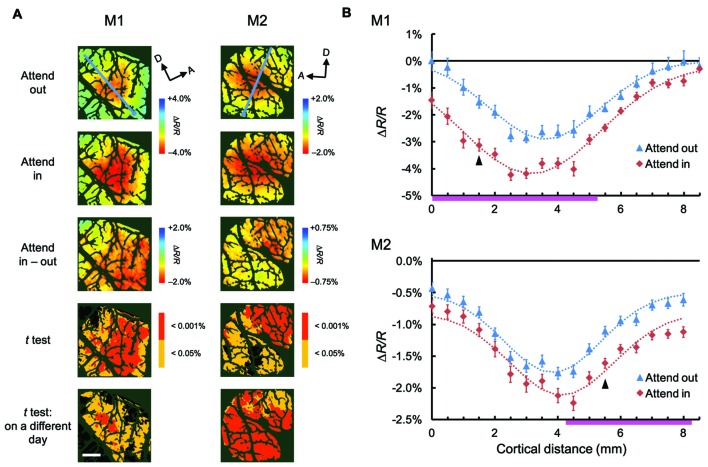
**Spatial distribution of attentional effects on global signals of hemodynamic responses. (A)** The first two rows show maps of stimulus-evoked reflectance changes averaged across all stimuli for both animals (global signals; M1, left; M2, right) under attend-out (first row) and attend-in (second row) conditions. The third row shows the difference maps between attend-in and attend-out conditions. The fourth row shows statistical maps in which regions with a significant difference in reflectance change between attend-in and attend-out conditions are color-coded according to the level of significance (*P* value). Regions exhibiting a larger decrease in reflectance under attend-in and attend-out conditions are colored in yellow/red and cyan/blue, respectively. The bottom row shows other statistical maps constructed using the same method as in the fourth row, but from data acquired 10 days (left) and 2 days (right) later, to illustrate the reproducibility of the attentional enhancements. Scale bar represents 1 mm. **(B)** Plots of average reflectance changes in the imaging session shown in the first row of **(A)**, sampled along the lines across the cortical surface for both animals (M1, top; M2, bottom), nearly parallel to the lunate sulcus, as indicated by the cyan lines in the top panels of **(A)**. The sampling points were selected every 1 mm, starting from the ventral ends of lines, as indicated by the cyan dots in **(A)**, which correspond to the distance 0 on the *x* axis. Error bars represent SEM. The range of points showing significant differences between the two plots (attend-out vs. attend-in) is indicated by the pink thick lines along the *x* axis. For each attentional condition and animal, Gaussian curves were fitted to the plots and are shown as dotted lines (all *R*^2^ values were above 0.94). Arrowheads indicate the sampling points giving the maximal difference between the two fitted curves under attend-out and attend-in conditions for each animal.

To examine the attentional effects of stimulus-nonspecific global signals of hemodynamic responses, we averaged the responses across all stimulus conditions examined, in agreement with the definitions given by previous studies (Frostig et al., 1990; Malonek and Grinvald, 1996). Pixel-by-pixel statistical maps revealed that the averaged responses were enhanced in the attend-in condition relative to the attend-out condition in most of the stimulus-evoked regions (Figure 3A; fourth row, two-tailed *t* test, *P* < 0.05 and the lowest *P* in each region < 0.001, *n* = 76 trials for M1 and 135 trials for M2; bottom row, replicated in another imaging session, *n* = 124 trials for M1 and 161 trials for M2). The attentional enhancement was not always simply additive or multiplicative. In fact, in some cases, greater enhancements were biased toward the ventral regions from the peak of the response under the attend-out condition (closer to the foveal representation) in M1 and toward the dorsal region (further from the foveal representation) in M2 (Figure 3B). In summary, these data indicate that spatial attention enhances global signals of hemodynamic responses in V4 and that these enhancements are biased from the centers of the responses. The latter may be due to uneven attention in the stimulus. Because we determined the size of stimulus patches in such a manner as to activate multiple stimulus-selective functional domains, the patches might have been too large for a single focus of attention. Therefore, the monkeys might have attended to a part of the patch to perform the task.

**Figure 4:**
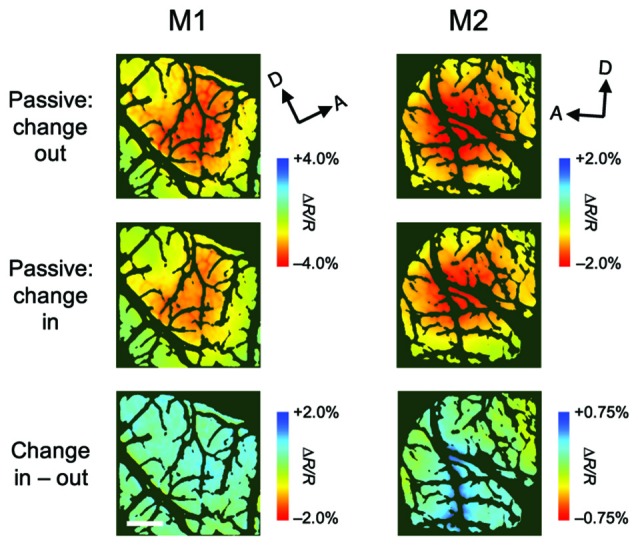
**Stimulus-evoked hemodynamic responses in passive viewing conditions.** These maps were obtained from a control experiment in which, in alternating blocks of trials, the color of the patch either outside or inside the population receptive field (pRF) of the imaged region changed (change-out and change-in, respectively), as the attention task, but the monkeys only received a reward after fixation without saccades. Therefore, the monkeys were not required to attend to the patches. All conventions are as in Figure 3.

Although attentional enhancement was observed prior to the color change of the patch (Figures 1C, 2C), it is possible that most of the observed enhancements were due to exogenous spatial attention, which might be involuntarily drawn to the color change (Carrasco, 2011). To determine the effect of such exogenous attention on the observed enhancement, we conducted a control experiment in which the monkey performed a passive viewing task while two patches were presented, followed by a color change in a patch either inside the pRF (change-in) or outside the pRF (change-out), as in the covert attention task (Figure 4). The difference in hemodynamic responses between change-in and attend-out conditions did not reach statistical significance at any pixel (two-tailed *t* test, *P* < 0.05 and the lowest *P* in each region, *n* = 215 trials for M1 and 124 trials for M2), suggesting that exogenous attention was not responsible for the enhancement of hemodynamic responses.

### Attentional Effects on Stimulus-Evoked Hemodynamic Responses of V4 Functional Domains

**Figure 5:**
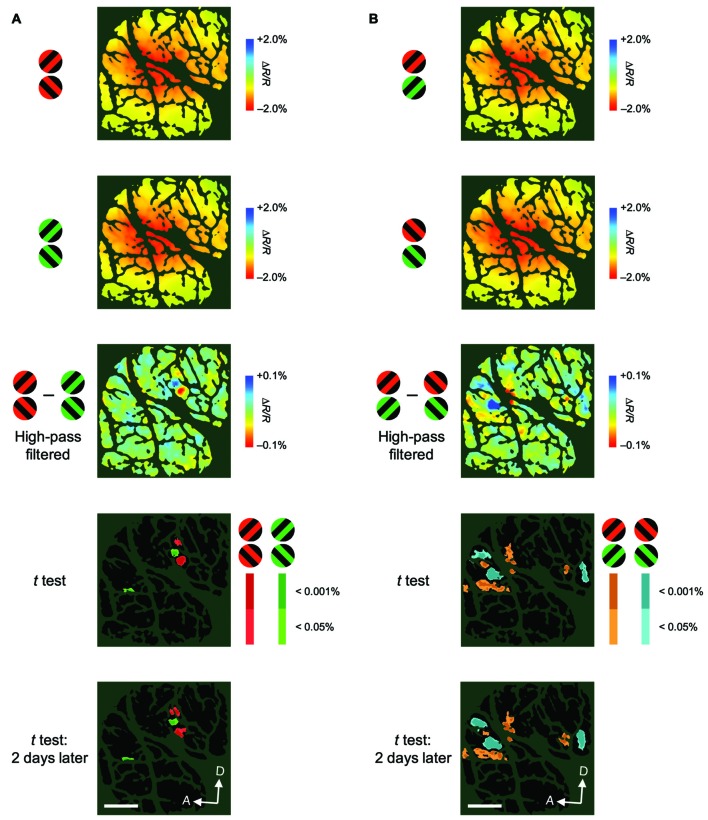
**Examples of color-preferring/orientation-preferring domains revealed in a difference map using high-pass filtered images. (A)** The first two panels show maps of reflectance changes evoked by patches of red/black (first) and green/black (second) gratings, averaged across two attentional conditions for M2. The third panel shows a difference map in response to red/black minus green/black gratings, constructed from high-pass filtered and blank-subtracted images. The fourth panel shows a statistical map in which regions exhibiting significant differences in their responses to red/black and green/black gratings are color-coded. Colored areas (red or green) indicate significantly larger response to either red/black or green/black gratings, according to the key shown on the right. The brightness of the color indicates the significance level: *P* < 0.05 (dark) and *P* < 0.001 (bright; two-tailed *t* test, *n* = 135 trials). The bottom panel shows another statistical map constructed with the same method as the fourth panel, but from data acquired 2 days later, to indicate the reproducibility of the domains (*n* = 161 trials). **(B)** The first two panels show maps of reflectance changes evoked by patches of gratings tilted 45° (first) and 135° (second) counterclockwise from the horizontal, averaged across two attentional conditions for M2. The third panel shows a difference map in response to 45°–135° gratings, constructed from high-pass filtered images. The fourth panel shows a statistical map in which regions exhibiting a significant difference in their responses to 45° and 135° gratings are color-coded (two-tailed *t* test, *n* = 135 trials). The bottom panel shows another statistical map constructed from data acquired 2 days later (*n* = 161 trials). Scale bar represents 1 mm.

**Figure 6:**
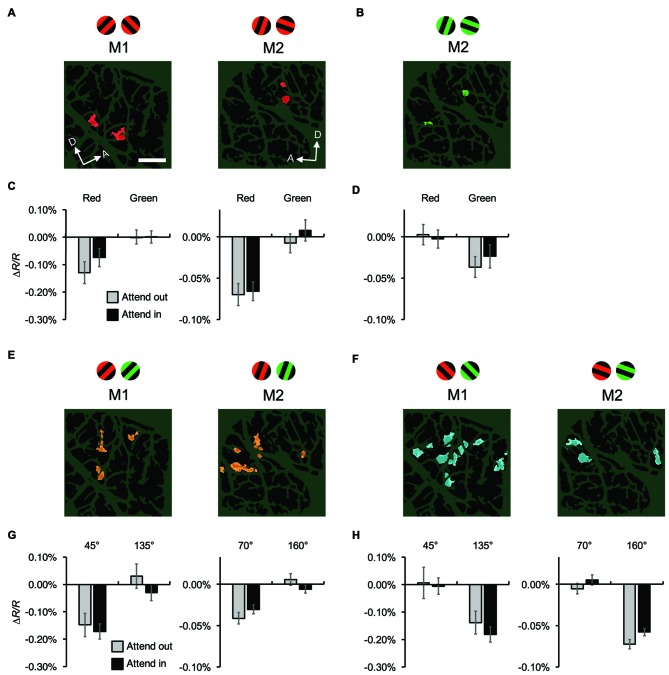
**Attentional effects on stimulus-specific hemodynamic responses of functional domains. (A,B)** Red-preferring **(A)** and green-preferring **(B)** domains revealed in statistical maps, as indicated in Figure 4, for both monkeys. The stimuli preferred by the domains are indicated above the maps. For M1, no green-preferring domain was observed in the imaged region. **(C,D)** Average reflectance changes of red-preferring **(C)** and green-preferring **(D)** domains, evoked by stimuli with preferred and non-preferred colors under different attentional conditions. **(E,F)** Orientation-preferring domains revealed in statistical maps for both monkeys. As described in “Materials and Methods” Section, the orientations of stimuli differed between monkeys: 45° and 135° counterclockwise from the horizontal for M1, 70° and 160° for M2. **(G,H)** Average reflectance changes of orientation-preferring domains, evoked by stimuli with preferred and non-preferred orientations under different attentional conditions. For visualization purposes, in **(C,D,G,H)**, we set the average reflectance changes of the non-preferred stimulus feature under the two attentional conditions to 0% of Δ*R/R*. Scale bar represents 1 mm.

Next, we examined the attentional effects on the stimulus-specific mapping signals of hemodynamic responses. On a trial-by-trial basis, maps of hemodynamic responses were spatially low-pass filtered and subtracted from the original map to remove global signals (see “Materials and Methods” Section). After blank subtraction, we calculated difference maps between two different color conditions (red vs. green) to generate color preference maps (Figure 5A), and between two orthogonal orientation conditions to generate orientation preference maps (Figure 5B). Using the same methodology, we successfully visualized mapping signals and identified color-preferring and orientation-preferring domains in V4 (Tanigawa et al., 2010). Cortical regions that repeatedly exhibited a significant preference for a particular color or orientation in multiple imaging sessions were regarded as functional domains for analysis (see “Materials and Methods” Section). We extracted four types of functional domains: red- and green-preferring domains for both monkeys, 45°- and 135°-preferring domains for M1, and 70°- and 160°-preferring domains for M2, and averaged hemodynamic response for each type of domains, respectively (Figure 6). Two-way ANOVAs in which one factor was the stimulus type (preferred vs. non-preferred stimulus feature) and the other factor was the attentional condition (attend-in vs. attend-out) revealed that for any type of domain, there was no significant difference in responses between the attentional conditions and no interaction between the two factors (*P* > 0.05, *n* = 76 trials for M1 and 135 trials for M2). Of course, as expected from the definitions of domains, there were significant differences in responses between preferred and non-preferred stimulus features for all types of domains, regardless of the attentional conditions (*P* < 0.0005). These results indicate that there is no attentional effect, at least on the amplitude of stimulus-specific hemodynamic responses of functional domains.

## Discussion

In this study, we used optical imaging techniques to reveal the spatial distribution of attentional modulation in macaque V4 at columnar resolution. We found that stimulus-evoked but stimulus-nonspecific global components of hemodynamic responses (global signals) were enhanced by spatial attention. The enhancements were not simply additive or multiplicative, and the peak of enhancement was slightly shifted from the peak of stimulus-evoked responses. We visualized feature-preferring domain activities (mapping signals) by removing the global signals with spatial filtering and differential mapping and found that the feature selectivity of domain activities was not affected by spatial attention. Given these results, we will propose a relationship between global signals and domain activities involved in normalization models of attention (Lee and Maunsell, 2009; Reynolds and Heeger, 2009).

### Neural Correlate of CBV-Based Hemodynamic Responses

In this study, we mapped stimulus-evoked hemodynamic responses by measuring light reflectance changes under 570 nm illumination. Light at this wavelength is equally absorbed by oxyhemoglobin and deoxyhemoglobin (an isosbestic wavelength, Prahl, 1998); therefore, the reflectance from the cortex provides a measure of local cortical tissue hemoglobin concentration, i.e., CBV. CBV-based hemodynamic signals had been thought to be more closely related to evoked local field potentials (LFPs) than to spiking activities (Logothetis et al., 2001; Sheth et al., 2003; Nemoto et al., 2004). Recently, Sirotin and co-workers have revealed that hemodynamic signals contain substantial task-related but stimulus-independent components that are not linked to neural activity, such as LFP and spiking activity (Sirotin and Das, 2009; Sirotin et al., 2012). Instead, stimulus-related components of CBV-based hemodynamic signals, which are obtained by removing task-related components with blank subtraction, are correlated linearly with spiking activities (the median determination coefficient *R^2^* = 0.83) more effectively than with LFP measurements (Cardoso et al., 2012; Lima et al., 2014). In this study, we also used blank subtraction to extract stimulus-related components in different attentional conditions. Therefore, the maps of CBV-based hemodynamic responses that we obtained should reflect the spatial distribution of stimulus-evoked spiking activities.

### Functional Significance of Global Signals in Spatial Attention

We revealed that stimulus-nonspecific global signals of CBV-based hemodynamic responses are subject to attentional enhancement. In primary visual cortex (V1), cortical spread of global signals evoked by a point-like, spatially focused stimulation, known as cortical point spread, is typically more than several millimeters (Frostig et al., 1990; Sirotin et al., 2009). The amplitude of these signals was more than 10 times larger than the amplitude of stimulus-specific mapping signals (Frostig et al., 1990; Fukuda et al., 2005), but less stable (Tanigawa et al., 2010). For the purpose of mapping stimulus preference in the cortex, such as orientation preference, global signals are often regarded as less important and removed by calculating differences between different stimulus conditions (Frostig et al., 1990) and/or by using spatial high-pass filtering (Tsunoda et al., 2001; Tanigawa et al., 2010). Our results shed light on the functional significance of global signals.

Normalization models of attention (Lee and Maunsell, 2009; Reynolds and Heeger, 2009) have been proposed to describe the effects of attention on sensory responses with divisive normalization (Heeger, 1992) and can successfully account for most of the known modulatory effects of attention (Carandini and Heeger, 2012). In this model, the initial stimulus-specific activation of neurons (stimulus drive) is suppressed (or normalized) by the summed activity of a broadly tuned pool of neighbor neurons (normalization pool). The normalization pool is driven by the stimulus drive and works for stimulus non-specific suppression. In a normalization model of attention (Reynolds and Heeger, 2009), top-down attention enhances the stimulus drive before normalization and, as a result, also enhances the summed activity of normalization pool. In another normalization model of attention (Lee and Maunsell, 2009), however, attention enhances the summed activity of the normalization pool, but not through the stimulus drive. In any of these models, the characteristics of the normalization pool for attention are very similar to those of global signals observed in our study. Suppression by the normalization pool is thought to be mediated by local inhibitory interneurons (Lee and Maunsell, 2009); fast-spiking neurons, which are presumed to be inhibitory interneurons (McCormick et al., 1985; Nowak et al., 2003), have higher spontaneous firing rates and larger attention-dependent increases in firing rate than regular-spiking neurons, which are presumed to be excitatory (Mitchell et al., 2007). As we discussed, CBV-based hemodynamic responses are well correlated with spiking activities and are therefore sensitive to local increases in firing rate. These speculations might suggest that global signals of CBV-based hemodynamic responses reflect the spatial distribution of normalization pool.

An optical imaging study using VSD also revealed the spatial distribution of attentional modulation in macaque V1 (Chen and Seidemann, 2012). The authors revealed that attentional modulation of VSD imaging signals is stimulus-nonspecific and acts in an additive manner, in which the spatially uniform baseline component of VSD signals is enhanced but neither the amplitude nor the shape of the Gaussian component is affected by attention. Given that VSD signals are linearly related to membrane potentials (Salzberg et al., 1973), the attentional enhancement of VSD baseline signals might reflect top-down inputs to a population of neurons in the imaged region (Chen and Seidemann, 2012). It would be interesting to examine the spatial distributions of attentional modulation using both VSD and CBV-based optical imaging in the same experimental conditions, in order to understand the spatial relationship between attentional top-down inputs and resultant spiking activities.

### Possible Attentional Effects on the Responses of Functional Domains

In contrast to global signals, stimulus-specific hemodynamic responses (mapping signals), which are extracted by removing global signals with spatial filtering and exhibit domain-like structures, are not significantly affected by spatial attention. Because mapping signals are by definition stimulus-specific, these signals seem to correspond to the stimulus drive in normalization models of attention. If so, our results might support the Lee and Maunsell’s normalization model of attention, which supposes that top-down attention controls the normalization pool without the mediation of the stimulus drive. To evaluate the validity of the models in detail using optical imaging, it would be necessary to determine whether the response to stimuli with various contrasts under different attentional conditions follows the models.

Besides changes in response amplitude, there are other ways in which attention can affect neuronal processing, such as enhanced gamma-band synchronization (Fries et al., 2001, 2008), reduced trial-to-trial neuronal variability (Mitchell et al., 2007), and reduced interneuronal correlation in trial-to-trial fluctuations (noise correlation; Cohen and Maunsell, 2009; Mitchell et al., 2009). These types of attentional modulation might affect the responses of functional domains. Indeed, our preliminary results showed that attention can enhance the correlation in hemodynamic responses among stimulus-evoked domain-like structures (Zhang et al., 2016). In addition, it is well known that feature-based attention, i.e., attention directed at a specific stimulus feature (e.g., orientation, direction, or color), also modulate neuronal processing (Treue et al., 1999; McAdams and Maunsell, 2000; Cohen and Maunsell, 2011a). This type of attention might affect the stimulus-specific hemodynamic responses, and should be further examined using optical imaging methods.

## Author Contributions

HT and AWR designed the experiment, HT performed the experiment and analysis, GC assisted HT with experimental procedures, HT wrote the manuscript, HT, GC and AWR discussed and reviewed the manuscript.

## Funding

This work was supported by a Grant-in-Aid for Scientific Research (15K01851) from Japan Society for the Promotion of Science (JSPS) to HT; grants from National Natural Science Foundation of China (31471052), Fundamental Research Funds for the Central Universities (2015QN81007), Zhejiang Provincial Natural Science Foundation of China (LR15C090001) to GC; and grants from Chinese NSF No. 81430010, Chinese 863 Grant No. 2015AA020515, US National Institutes of Health grant EY11744, Vanderbilt Vision Research Center and Vanderbilt University Center for Integrative and Cognitive Neuroscience to AWR.

## Conflict of Interest Statement

The authors declare that the research was conducted in the absence of any commercial or financial relationships that could be construed as a potential conflict of interest.
